# Effect of Sodium Nitrite on Ischaemia and Reperfusion-Induced Arrhythmias in Anaesthetized Dogs: Is Protein S-Nitrosylation Involved?

**DOI:** 10.1371/journal.pone.0122243

**Published:** 2015-04-24

**Authors:** Mária Kovács, Attila Kiss, Márton Gönczi, Gottfried Miskolczi, György Seprényi, József Kaszaki, Mark J. Kohr, Elizabeth Murphy, Ágnes Végh

**Affiliations:** 1 Department of Pharmacology and Pharmacotherapy, Faculty of Medicine, University of Szeged, Szeged, Hungary; 2 Department of Medical Biology, Faculty of Medicine, University of Szeged, Szeged, Hungary; 3 Institute of Surgical Research, Albert Szent-Györgyi Medical Center, University of Szeged, Szeged, Hungary; 4 Systems Biology Center, National Heart Lung and Blood Institute, National Institutes of Health, Bethesda, Maryland, United States of America; University of Oxford, UNITED KINGDOM

## Abstract

**Background and Purpose:**

To provide evidence for the protective role of inorganic nitrite against acute ischaemia and reperfusion-induced ventricular arrhythmias in a large animal model.

**Experimental Approach:**

Dogs, anaesthetized with chloralose and urethane, were administered intravenously with sodium nitrite (0.2 µmolkg^-1^min^-1^) in two protocols. In protocol 1 nitrite was infused 10 min prior to and during a 25 min occlusion of the left anterior descending (LAD) coronary artery (NaNO_2_-PO; n = 14), whereas in protocol 2 the infusion was started 10 min prior to reperfusion of the occluded vessel (NaNO_2_-PR; n = 12). Control dogs (n = 15) were infused with saline and subjected to the same period of ischaemia and reperfusion. Severities of ischaemia and ventricular arrhythmias, as well as changes in plasma nitrate/nitrite (NOx) levels in the coronary sinus blood, were assessed throughout the experiment. Myocardial superoxide and nitrotyrosine (NT) levels were determined during reperfusion. Changes in protein S-nitrosylation (SNO) and S-glutathionylation were also examined.

**Key Results:**

Compared with controls, sodium nitrite administered either pre-occlusion or pre-reperfusion markedly suppressed the number and severity of ventricular arrhythmias during occlusion and increased survival (0% vs. 50 and 92%) upon reperfusion. There were also significant decreases in superoxide and NT levels in the nitrite treated dogs. Compared with controls, increased SNO was found only in NaNO_2_-PR dogs, whereas S-glutathionylation occurred primarily in NaNO_2_-PO dogs.

**Conclusions:**

Intravenous infusion of nitrite profoundly reduced the severity of ventricular arrhythmias resulting from acute ischaemia and reperfusion in anaesthetized dogs. This effect, among several others, may result from an NO-mediated reduction in oxidative stress, perhaps through protein SNO and/or S-glutathionylation.

## Introduction

Inorganic nitrite and nitrate, the nautal metabolites of nitric oxide (NO) have been considered for a long time as inert molecules without further biological activity. More recently this concept has been challenged, due to the accumulating experimental and clinical evidence, which shows that these molecules may play an important physiological role in mediating the biological effects of nitric oxide [1, 2]. It has been recognized that these oxidative metabolites of NO are readily reduced back to NO, especially under hypoxic or anoxic conditions, when a drop in pH and oxygen tension promotes reductive processes [3]. This enzyme-independent NO formation might be of particular importance during ischaemia when in the absence of oxygen, the NO production by nitric oxide synthase (NOS) enzymes becomes limited. Thus nitrite can serve as a fundamental natural store of NO that serves to maintain cardiac function under ischaemic conditions [4, 5].

There is strong evidence that increasing NO bioavailability, for example, by the administration of organic nitrites and nitrates, which are thought to act as NO donors [6], suppresses the generation of arrhythmias that results from acute ischaemia and reperfusion in dogs [7–9] and also in humans [10, 11]. Although the precise mechanism of this antiarrhythmic effect is not fully elucidated, clinical studies have revealed that nitroglycerin causes electrophysiological alterations in the human heart [10], and reduces ectopic activity during acute myocardial infarction [11]. More recently we have reported that the antiarrhythmic effect of sodium nitroprusside is associated with the preservation of gap junctional function [9]. Another way to increase NO availability is via ischaemic preconditioning. We have evidence that preconditioning induced either by brief ischaemic episodes [12], cardiac pacing [13–15] or heavy physical exercise [16], stimulates the generation of NO in vascular endothelial cells and also in cardiac myocytes [17], and this NO plays an important trigger and mediator role in both the early and delayed cardioprotective (antiarrhythmic) effects of preconditioning [13–15, 18].

Nitric oxide exerts its cardioprotective effects via cGMP-dependent and cGMP-independent pathways [3, 19, 20]. For example, in dogs the antiarrhythmic effect of preconditioning was completely abolished by the inhibition of the NO-cGMP pathway [21]. On the other hand, we hypothesize that the preservation of NO availability following preconditioning and the subsequent reduction in superoxide production, which plays an essential role in the antiarrhythmic effect of preconditioning [22], could also contribute to the protection, at least in part, via cyclic-GMP-independent mechanisms, such as protein S-nitrosylation and S-transnitrosylation [23–25]. These are thought to provide protection by preventing the oxidation of proteins during the initial phase of reperfusion, when a burst of reactive oxygen species (ROS) usually occurs [26]. S-nitrosylation (SNO) is the covalent attachment of an NO moiety to a cysteine residue of proteins. Formation of SNO allows for storage and transport of NO [27], as well as the regulation of several cardiac functions by NO, including the regulation of cardiac ion channels [28, 29], mitochondrial respiration [30] and ROS generation [23]; all of these may be connected to arrhythmogenesis [31].

Although a number of studies have shown that inorganic nitrites and nitrates, as natural reservoirs for NO, may regulate cardiovascular function and provide cardioprotection [32–34], there is a lack of information how these modify the severity of arrhythmias. Therefore, we designed studies in anaesthetized dogs to examine whether sodium nitrite provides protection against the acute ischaemia and reperfusion-induced severe ventricular arrhythmias, and if so, whether this effect involves protein SNO. Preliminary accounts of these studies were given at the ISHR World Congress in Kyoto [35] and at the meeting on ‘Approaching the Clinic: Nitrite and Nitrate Pathophysiology and Therapy’ in Atlanta, USA [36].

## Methods

### Ethics statement

The origin and upkeep of these dogs were in accord with Hungarian law (XXVIII, chapter IV, paragraph 31) regarding large experimental animals, which conforms with the *Guide for the Care and Use of Laboratory Animals* published by the US National Institutes of Health (NIH Publication No. 85–23, revised 1996), and conformed to the Directive 2010/63/EU of the European Parliament. The protocols have been approved by the Ethical Committee for the Protection of Animals in Research of the University of Szeged, Szeged, Hungary (approval number: I-74-5-2012) and by the Department of Animal Health and Food Control of the Ministry of Agriculture and Rural Development (authority approval number XIII/1211/2012).

### Surgical procedures

Inbred mongrel dogs of either sex, and with a mean body weight in excess of 17 kg (mean 24 ± 3 kg) were anaesthetized with an intravenous bolus injection of pentobarbitone (0.5 mg kg-1 i.v., Euthanyl Bimeda-MTC Animal Health Inc.) and all efforts were made to minimize suffering. Prior to the studies the dogs were housed in an animal room (temperature:10–20°C, humidity: 40–70%, lightening: 12 h per day, 2 animals per pen), for at least two weeks and fed a standard diet with free access to water. Food was withdrawn 24 h before anaesthesia.

After catheterization of the right femoral vein, the anaesthesia was maintained with the intravenous administration of a mixture of chloralose and urethane (60 and 200 mg kg-1, respectively; Sigma, St. Louis, MO, USA). The depth of anaesthesia was monitored by the examination of the cornea and pain reflexes as well as by the measurement of blood pressure, and when it was necessary, a further bolus injection of the anaesthetic was given. The dogs were ventilated with room air using a Harvard respirator at a rate and volume sufficient to maintain arterial blood gases within normal limits [12, 22]. Body temperature was measured from the mid-esophagus and maintained at 37 ± 0.5°C.

Catheters (Cordis F4) were inserted into the right femoral artery for monitoring arterial blood pressure (systolic, diastolic, and mean), and into the left ventricle (LV) for the measurement of LV systolic and end-diastolic pressures and LVdP/dt. All pressure parameters, together with a chest lead electrocardiogram, were measured with a Plugsys Hemodynamic Apparatus (Hugo Sachs Electronics, Germany) and recorded on a Graphtec Thermal Array Recorder (Hugo Sachs Electronics, Germany).

A thoracotomy was performed at the left fifth intercostal space and the anterior descending branch of the left coronary artery (LAD) prepared for occlusion just proximal to the first main diagonal branch. Through the right jugular vein, a catheter was positioned into the coronary sinus to obtain blood samples for the measurement of plasma nitrate/nitrite (NOx) levels. In some control and treated dogs, the left circumflex (LCX) coronary artery was also prepared to measure coronary blood flow (CBF; ml min^-1^) by means of a transit time Doppler flow probe (Hugo Sachs Electronics, Germany).

The severity of myocardial ischaemia was assessed by the measurement of changes in the degree of electrical activation and in epicardial ST-segment using a mapping electrode positioned within the ischaemic region [37]. Signals were collected from 31 unipolar electrodes (inter-electrode distance; 2 mm) at a frequency of 1 kHz, stored on a computer and analyzed offline by creating activation and ST maps. Changes in epicardial activation were assessed as a time delay between the first and a last point activated under the electrode and expressed as the total activation time (TAT) in milliseconds. Elevations of epicardial ST-segment, recorded in each minute from the unipolar electrodes were averaged and expressed in mV.

Ventricular arrhythmias were evaluated according to the Lambeth Conventions [38] with a modification as previously outlined [12]. Thus, the total number of ventricular premature beats (VPBs), the incidence and the number of episodes of ventricular tachycardia (VT; defined as a run of four or more consecutive VPBs at a rate faster than the resting heart rate), and the incidence of ventricular fibrillation (VF) were assessed during the occlusion period. During reperfusion, only the incidence of VF (which is a final event in this species) was determined. Dogs that were still alive 2 min after reperfusion (the end of the study) were considered to be survivors.

### Measurement of plasma nitrate/nitrite (NOx) levels

These were performed as described previously [22]. Plasma nitrate/nitrite (NO_x_) concentrations were determined by means of the Griess reaction in blood samples taken from the coronary sinus at various time intervals as described in the protocol. After sample preparation, the absorbance of the azo compound was measured spectrophotometrically at a wavelength of 540 nm and the total nitrate/nitrite (NO_x_) concentration (μmol L^-1^) was determined using a standard calibration curve of NaNO_2_ and NaNO_3_ (Sigma St Louis, MO, USA).

### Assessment of superoxide production

This has also been described previously [22]. In brief, tissue blocks (size: 0.5 x 0.5 x 2.0 cm), excised from the ischaemic area within 2 min of the reperfusion, were embedded in optimal cutting temperature compounds. Cryosections (20 μm) were produced and stained with dihydroethidium (Sigma, St Louis, MO, USA). A negative control was obtained by blocking the reaction with N-acetyl-L-cysteine (100 mmol L^-1^, Sigma St Louis, MO, USA). Both from the stained and negative control samples 10–15 serial images were captured by a confocal laser scanning microscope (Olympus FV1000). The intensity of the fluorescent signals was analyzed by ImageQuant software (Molecular Dynamics) and expressed in arbitrary units.

### Determination of peroxynitrite production

This was performed by the measurement of nitrotyrosine formation using Western immunoblot as described previously [22]. In brief, after tissue preparation (homogenization and centrifugation) the supernatant was collected and the protein concentration was determined by the Lowry method. Following electrophoresis the proteins were transferred onto PVDF membrane. After incubation with a monoclonal anti-nitrotyrosine antibody (MAB5404, Chemicon, USA), and horseradish peroxidase-conjugated rabbit anti-mouse IgG (P0161, Dakocytomation, Denmark) as the secondary antibody, the membrane was developed with an enhanced chemiluminescence kit (ECL Plus, GE Healthcare, UK), exposed to X-ray film and scanned. ImageJ (NIH, Bethesda, MD) was used to determine the density of nitrotyrosine bands. Equal loading was controlled using Coomassie Blue staining.

### Assessment of protein S-nitrosylation and glutathionylation

Tissue homogenates from canine left ventricle were prepared as previously described [26], and protein concentrations were determined using the Bradford protein assay. The SNO-RAC protocol was used to examine protein SNO, as previously described [26]. Briefly, samples were incubated with 50 mmol L^-1^ n-ethylmaleimide for 20 minutes at 50°C in order to block non-modified (i.e., free) thiol groups. Samples were then incubated with thiopropyl sepharose resin (GE Healthcare, Piscataway, NJ) in the presence of 20 mmol L^-1^ ascorbate to reduce SNO, and rotated for four hours in the dark at room temperature. Resin-bound proteins were then subjected to trypsin digestion (sequencing grade modified; Promega, Madison, WI) overnight at 37°C. Resin-bound peptides were eluted in buffer containing (in mmol L^-1^): DTT (20), NH_4_HCO_3_ (10), and 50% methanol (v/v), and identified using liquid chromatography-tandem mass spectrometry. Mass spectrometry results were analyzed using Proteome Discoverer v1.3 software (Thermo Fisher Scientific) with the NIH six-processor MASCOT cluster search engine (http://biospec.nih.gov, version 2.3). Proteome Discoverer v1.3 software was also used to provide relative quantification of SNO levels via label-free peptide quantification.

Glutathionylation was examined using Western immunoblot. Following electrophoresis, proteins were transferred onto a nitrocellulose membrane, which was subsequently blocked with LI-COR blocking buffer (Lincoln, NE) containing 25 mmol L^-1^ n-ethylmaleimide. After this the membrane was incubated with an anti-glutathione antibody (Virogen, Watertown, MA), followed by incubation with a fluorescent secondary antibody (LI-COR). The membrane was imaged using a LI-COR Odyssey scanner. ImageJ (NIH, Bethesda, MD) was used to determine the density of glutathionylation bands.

### Experimental protocol

**Fig 1:**
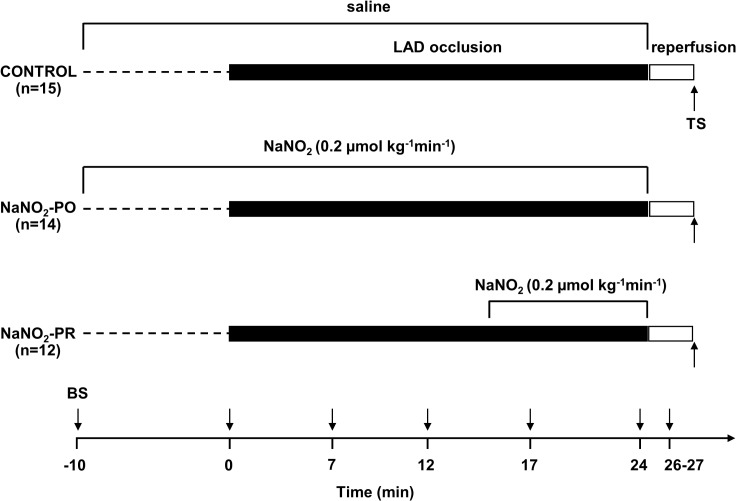
Experimental protocol. After surgery all animals were allowed to equilibrate for 20 min. Fifteen dogs (control group) were infused with pH 7.4 saline (rate: 0.5 ml min^-1^) via intravenous route over the entire observation period. In another two groups of dogs, sodium nitrite was administered in intravenous infusion, starting the infusion either prior to and maintained throughout the occlusion (NaNO_2_-PO; n = 14) or 10 min prior to reperfusion (NaNO_2_-PR; n = 12). In all groups, ischaemia was induced by a 25 min occlusion of the LAD, followed by rapid reperfusion. During the experiment, blood samples (BS) were collected at various time intervals from the coronary sinus for the assessment of plasma nitrate/nitrite (NOx) levels. Within 2 min of reperfusion tissue samples (TS) were taken from the ischaemic myocardium for further analyses.

The experimental protocol is illustrated in Fig 1. A total of 41 dogs were used and randomly divided into three groups. Each animal was subjected to a 25 min LAD occlusion followed by rapid reperfusion. Control dogs (C; n = 15) were infused with saline, commencing prior to and then throughout the 25 min occlusion period. The concentration of sodium nitrite has been selected on the basis of the study of Gonzalez et al., [39], and was administered by intravenous infusion at a concentration of 0.2 μmol kg^-1^ min^-1^ to two groups of dogs; in one group (NaNO_2_-PO; n = 14) the infusion was started 10 min prior to and maintained over the entire occlusion period, whereas in the other group (NaNO_2_-PR; n = 12), the infusion was given just 10 min prior to reperfusion. In dogs that survived the combined ischaemia and reperfusion insult, the hearts were stopped within 2 min with an overdose of the anaesthetic and myocardial tissue samples were collected from the ischaemic ventricular wall. In dogs that suddenly fibrillated on reperfusion, samples were quickly excised when the fibrillation had been observed. An additional six dogs served as sham-operated controls (not included in the protocol). These dogs were instrumented and infused locally with saline without subjection to ischaemia. At the end of the observation period the animals were euthanized with an excess of anaesthetic and myocardial samples were taken for further analyses.

In those dogs in which tissue samples were not taken (at least n = 5 dogs in each group), the heart was excised and the risk area was assessed by injecting Patent Blue V dye into the re-occluded artery using the same method that has been described in detail elsewhere [12].

### Statistical analysis

All data were expressed as mean ± SEM and the differences between means were compared by ANOVA for repeated measures and by one-way ANOVA as appropriate, using the Fisher post hoc, Bonferroni and Newman-Keuls multiple comparison tests. Ventricular premature beats and episodes of ventricular tachycardia were compared using the Kruskal-Wallis test. The incidence of arrhythmia (such as VT and VF) and survival from the combined ischaemia and reperfusion insult was compared by the Fisher Exact test. Differences between groups were considered significant when ^*^ *P* < 0.05.

## Results

### Haemodynamic effects of intravenous sodium nitrite and subsequent coronary artery occlusion

The administration of sodium nitrite resulted in a slight reduction in arterial blood pressure, LVEDP and in positive and negative LVdP/dt_max_ without substantial changes in heart rate and in mean CBF measured on the LCX artery (Table 1). When the LAD was occluded for 25 minutes, the haemodynamic changes were similar in all groups, except that in dogs infused with sodium nitrite, the increases in LVEDP were less pronounced than in the controls (Table 1). Compared to controls, the compensatory blood flow changes occurring on the LCX when the LAD was occluded were not modified by sodium nitrite. There were also no significant alterations in blood methemoglobin concentrations following sodium nitrite administration. These values increased from around 0.15 ± 0.03% measured at baseline to 0.20 ± 0.01% in controls, to 0.38 ± 0.03% in NaNO_2_-PO and to 0.26 ± 0.03% in NaNO_2_-PR groups by the end of the occlusion.

**Table 1:** The haemodynamic effects of NaNO_2_ infusion and coronary artery occlusion.

	SALINE				NaNO_2_-PO				CONTROL				NaNO_2_-PO				NaNO_2_-PR			
	Baseline		Max. change		Baseline		Max.Change		Baseline		Max. changeon occlusion		Baseline		Max. changeon occlusion		Baseline		Max. changeon occlusion	
**SABP (mm Hg)**	143	±4	-2	±2	132	±7	-8	±3^#^	141	±5	-11	±3^#^	124	±6	-13	±1^#^	150	±3	-13	±1^#^
**DABP (mm Hg)**	97	±3	-3	±1	89	±6	-6	±2^#^	98	±4	-11	±1^#^	85	±5	-9	±1^#^	104	±5	-11	±2^#^
**MABP (mm Hg)**	112	±3	-2	±1	103	±6	-6	±2^#^	110	±3	-11	±1^#^	98	±5	-11	±1^#^	119	±4	-12	±1^#^
**LVSP (mm Hg)**	145	±4	-2	±1	135	±6	-6	±2	146	±4	-15	±1^#^	124	±6	-12	±12^#^	154	±6	-15	±2^#^
**LVEDP (mm Hg)**	3.7	±0.3	0.3	±0.2	4.6	±0.3	-1.1	±0.3^#^ ^*^	3.7	±0.3	12.2	±1.3^#^	4.8	±0.3	6.4	±0.3^#^ ^*^	4.0	±0.2	6.2	±0.4^#^ ^*^
**+dP/dt (mm Hg s** ^**-1**^ **)**	3081	±109	-22	±20	2924	±77	-98	±48^#^ ^*^	3081	±149	-629	±94^#^	2846	±106	-525	±44^#^	3083	±89	-430	±53^#^ ^*^
**-dP/dt (mm Hg s** ^**-1**^ **)**	2600	±92	-12	±9	2257	±59	-112	±32^#^ ^*^	2556	±107	-588	±92^#^	2157	±77	-456	±48^#^	2219	±59	-342	±49^#^ ^*^
**HR (beats min** ^**-1**^ **)**	167	±3	0	±1	162	±4	4	±3	167	±4	-3	±2	164	±4	1	±2	167	±1	2	±2
**mCBF**_**LCX**_ **(ml min** ^**-1**^ **)**	30	±2	3	±1	32	±3	0	±1	28	±3	11	±1^#^	31	±3	12	±1^#^	29	±1	9	±2^#^

Values are mean ± SEM calculated from n = 12 to 15 experiments. Abbreviations: SABP: systolic blood pressure, DABP: diastolic blood pressure, MABP: mean arterial blood pressure, LVSP: left ventricular systolic pressure, LVEDP: left ventricular end diastolic pressure, HR: heart rate, mCBF_LCX_: mean coronary blood flow determined on the LCX coronary artery.

^#^ *P* < 0.05 vs. baseline value;

^*^ *P* < 0.05 vs. control group.

### Ventricular arrhythmias during coronary artery occlusion and reperfusion

**Fig 2:**
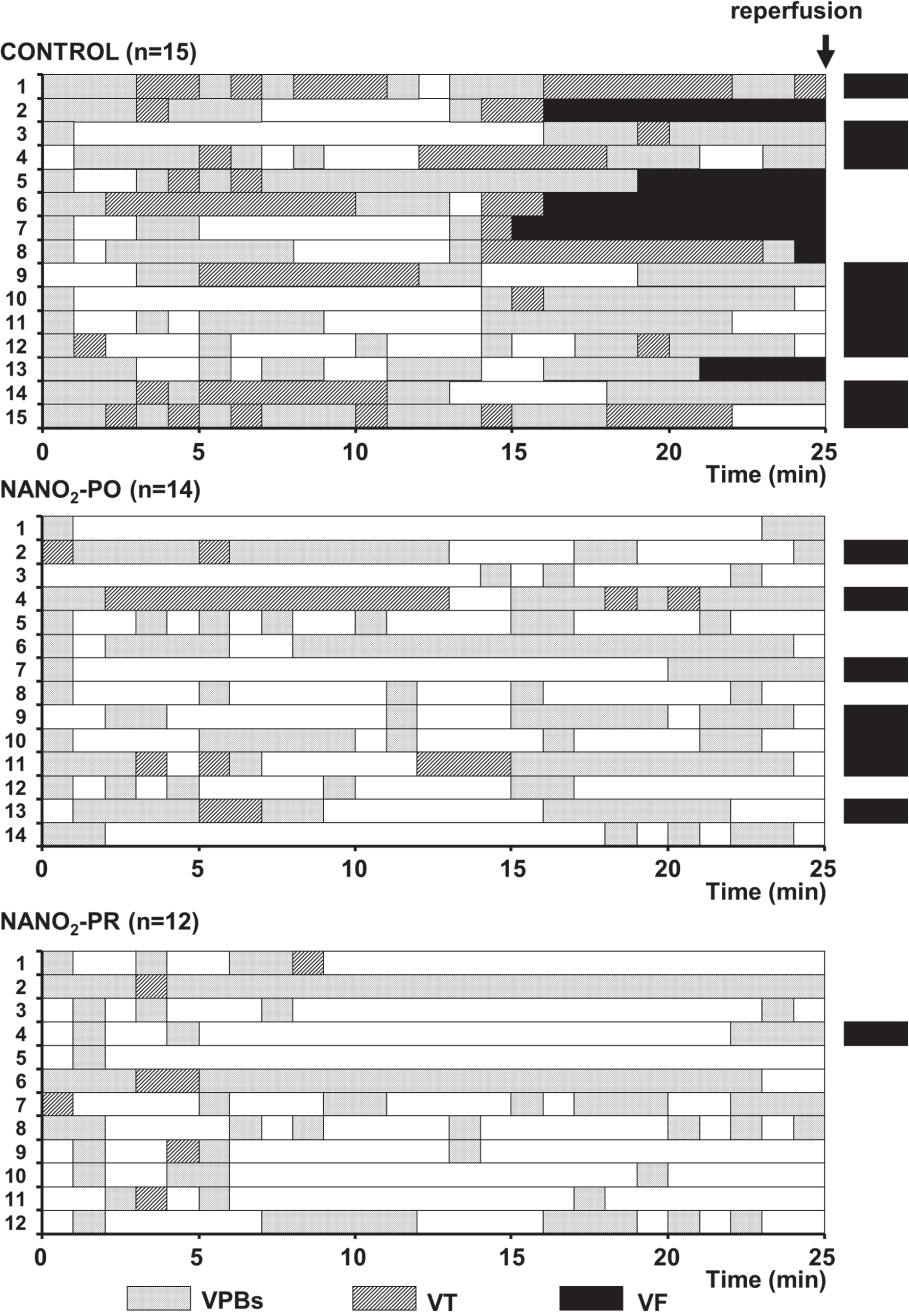
The distribution of ventricular arrhythmias following coronary artery occlusion and reperfusion. This is shown in each individual control dog and in dogs that were infused with sodium nitrite prior to and during the occlusion (NaNO_2_-PO group) and 10 min prior to reperfusion (NaNO_2_-PR group). The filled coulumns are the times during which the dog was in ventricular fibrillation (VF), the shaded columns show periods of ventricular tachycardia (VT) and the lightly stippled coulumns are periods during which ventricular premature beats (VPBs) were evident. VF on reperfusion is also shown by the black horizontal columns and this was the only arrhythmia assessed during the reperfusion phase. The results show that in the controls VF occurred in 6 dogs and that all the other dogs that survived the occlusion period fibrillated on reperfusion. Thus there were no survival in this group from the combined ischaemia and reperfusion insult. In contrast infusion of sodium nitrite either prior to and during the occlusion or just prior to reperfusion significantly reduced the severity of the occlusion-induced arrhythmias and increased survival on reperfusion. This later was particularly marked in dogs in which sodium nitrite was infused 10 min prior to reperfusion.

**Fig 3:**
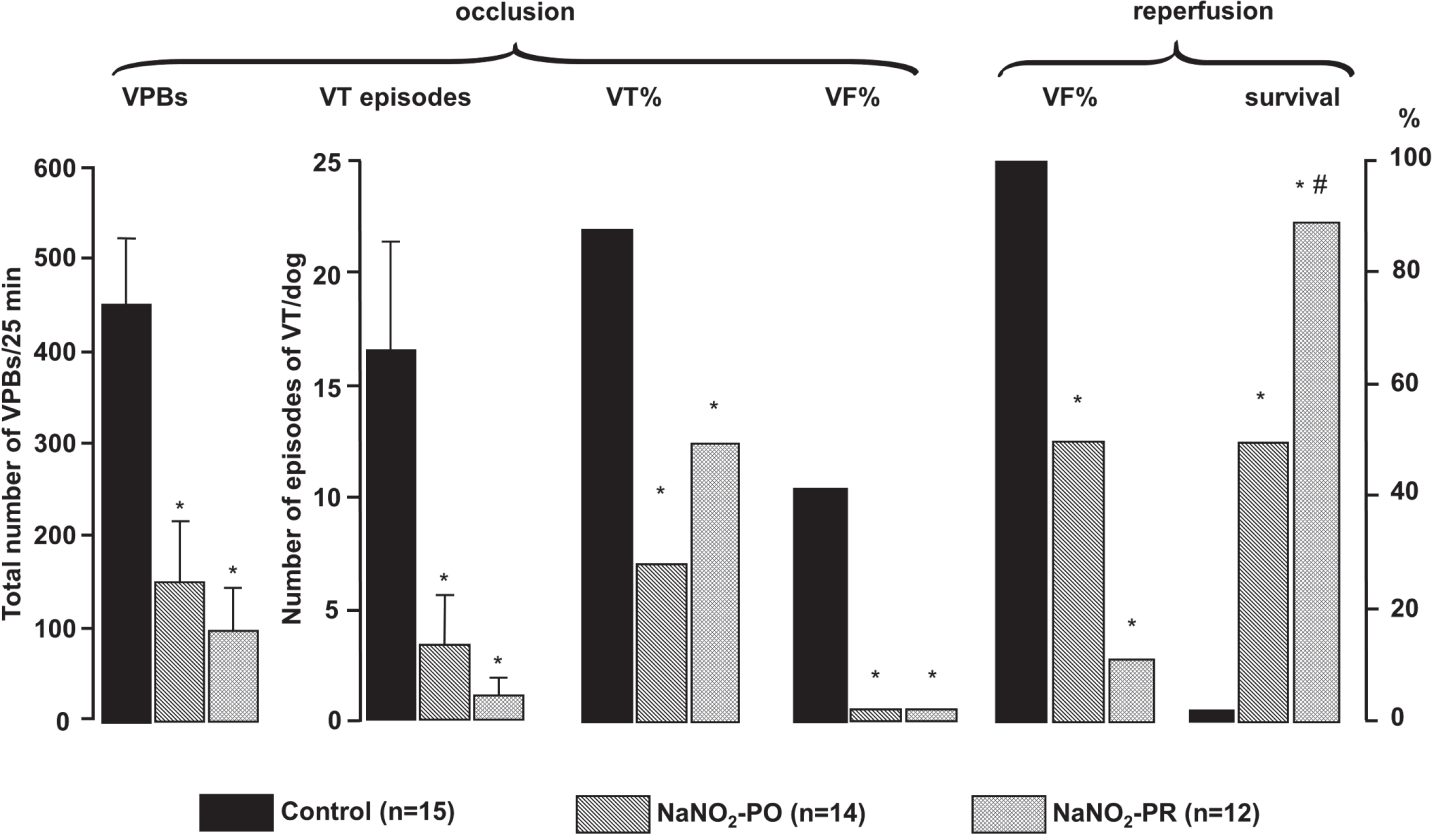
The severity of arrhythmias during a 25 min occlusion and reperfusion of the LAD in control dogs and in dogs infused with sodium nitrite. Both administration forms of sodium nitrite markedly reduced the total number of ventricular premature beats (VPBs), the incidence and the number of episodes of ventricular tachycardia (VT) during occlusion, and increased survival from the combined ischaemia and reperfusion insult. Values represent mean ± SEM. *P < 0.*05* vs. controls, ^*#*^ *P < 0*.*05* vs. NaNO_2_-PO group.

The distribution of various arrhythmia types during a 25 min occlusion and reperfusion (only VF was assessed) is shown in Fig 2 and the summary of the arrhythmia events is given in Fig 3. Compared with controls, sodium nitrite infused either prior to and continued over the entire occlusion period or given just 10 min prior to reperfusion resulted in marked reductions in the total number of VPBs (442 ± 91 vs. 157 ± 78 and 85 ± 42; *P* < 0.05), in the incidence (87% vs. 29 and 50%; *P* < 0.05) and the number of episodes of VT (15.5 ± 5.1 vs. 3.4 ± 2.4 and 0.6 ± 0.2; *P* < 0.05) during occlusion. Furthermore, no dog infused with sodium nitrite fibrillated during occlusion compared with 40% incidence of VF in the control animals. When the ischaemic myocardium was rapidly reperfused, all of the control dogs fibrillated thus there was no survival in this group compared with the sodium nitrite treated groups in which 50% (NaNO_2_-PO) and 92% (NaNO_2_-PR) of the animals survived.

### Ischaemia severity following coronary artery occlusion

**Fig 4:**
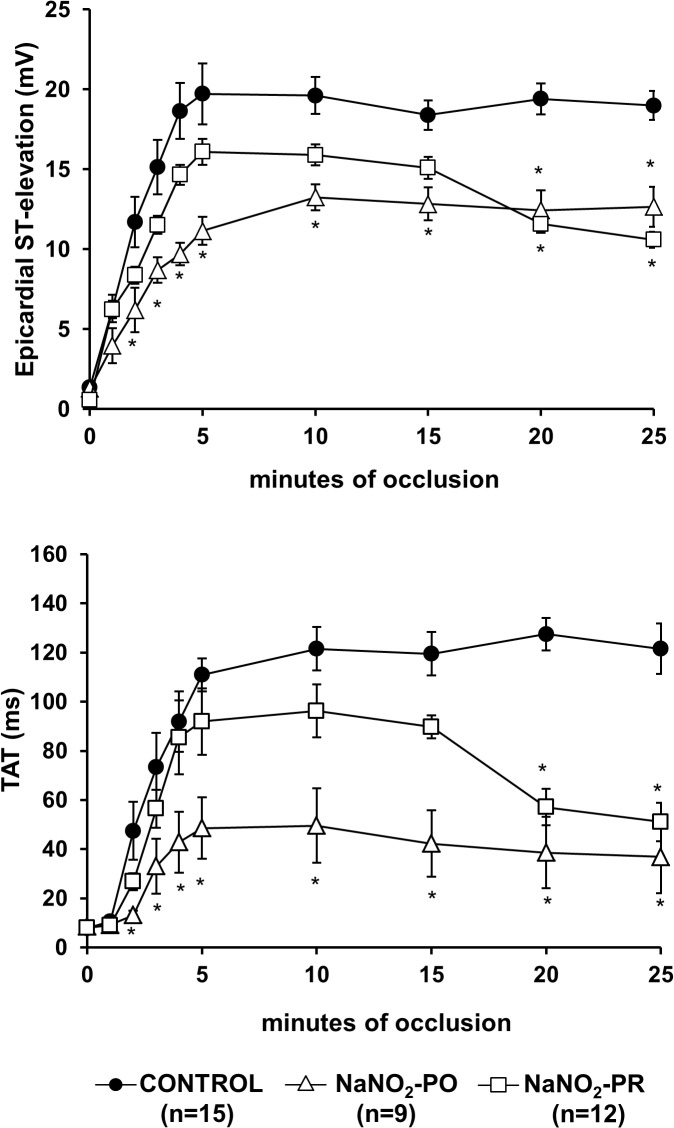
Changes in epicardial ST-segment (A) and in total activation time (TAT; B) during a 25 min occlusion of the anterior descending branch of the left coronary artery. In control dogs, both indices of ischaemia severity were markedly increased, especially during the initial 5 min of the occlusion. These changes were significantly less marked from the beginning of the occlusion in the NaNO_2_-PO group and from the 15 min of the occlusion in the NaNO_2_-PR group than in the controls. Values represent mean ± S.E.M. ^*^ *P* < 0.05 compared with the controls.

This was assessed by changes in epicardial ST-segment and TAT during a 25 min occlusion of the LAD (Fig 4A and 4B). In control dogs both parameters were markedly elevated during the initial 5 min of the occlusion and this was maintained over the entire occlusion period. Administration of sodium nitrite prior to occlusion significantly reduced these indices of ischaemia severity over the entire occlusion period. In contrast, compared with controls, in dogs infused with sodium nitrite 10 min prior to reperfusion, a significant reduction in epicardial ST (Fig 4A) segment and in TAT (Fig 4B) occurred only the last 7 min of the occlusion.

### Changes in plasma nitrite and nitrate as well as in total NOx levels during coronary artery occlusion and reperfusion

**Fig 5:**
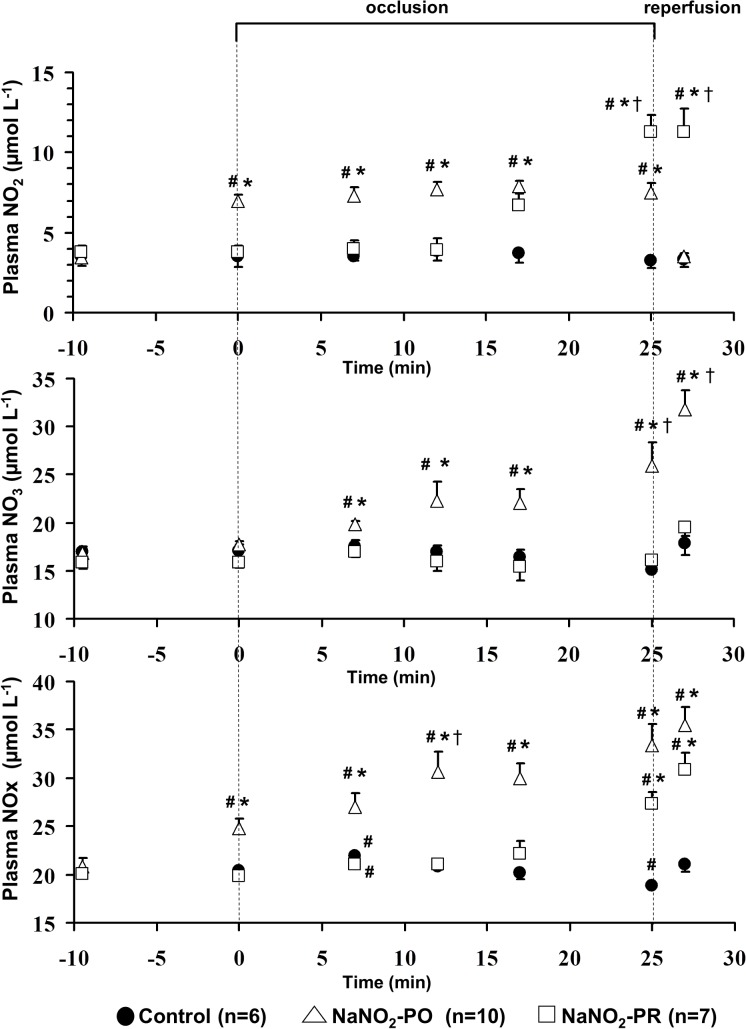
Changes in plasma nitrite and nitrate as well as total nitrite/nitrate (NOx) levels in the blood of the coronary sinus. In control dogs the level of NO metabolites was significantly reduced by the end of the occlusion period. Infusion of sodium nitrite before and during occlusion increased nitrite, nitrate and NOx concentrations, whereas when nitrite was administered 10 min prior to reperfusion the increase in NOx resulted only from the elevation of nitrite concentration. Reperfusion caused almost similar increases of NOx in all groups. Values represent mean ± SEM. *P < 0.*05* vs. controls, ^*#*^ *P < 0*.*05* vs. the initial baseline value and ^†^ *P < 0*.*05* vs. NaNO_2_-PO group.

These are shown in Fig 5. The plasma total NOx concentration in the coronary sinus blood was 21.36 ± 0.10 μmol L^-1^ as determined from 41 dogs at baseline. In control dogs, infusion of saline did not modify the plasma level of NO metabolites. However, when the LAD was occluded, the NOx levels, after a transient elevation occurring around the seventh min of ischaemia, were markedly reduced by the end of the occlusion period. Infusion of sodium nitrite prior to occlusion (NaNO_2_-PO group), resulted in significant increases in both nitrite and nitrate levels, and these were maintained or even further increased during occlusion. In contrast, in dogs infused with sodium nitrite 10 min prior to reperfusion (NaNO_2_-PR group), there was no substantial increase in nitrate levels up to the end of the occlusion. Reperfusion of the LAD resulted in almost similar step increases in plasma NOx levels in all groups, although the absolute concentration values were different among the groups.

### The effect of sodium nitrite on the reperfusion-induced myocardial superoxide and nitrotyrosine formation

**Fig 6:**
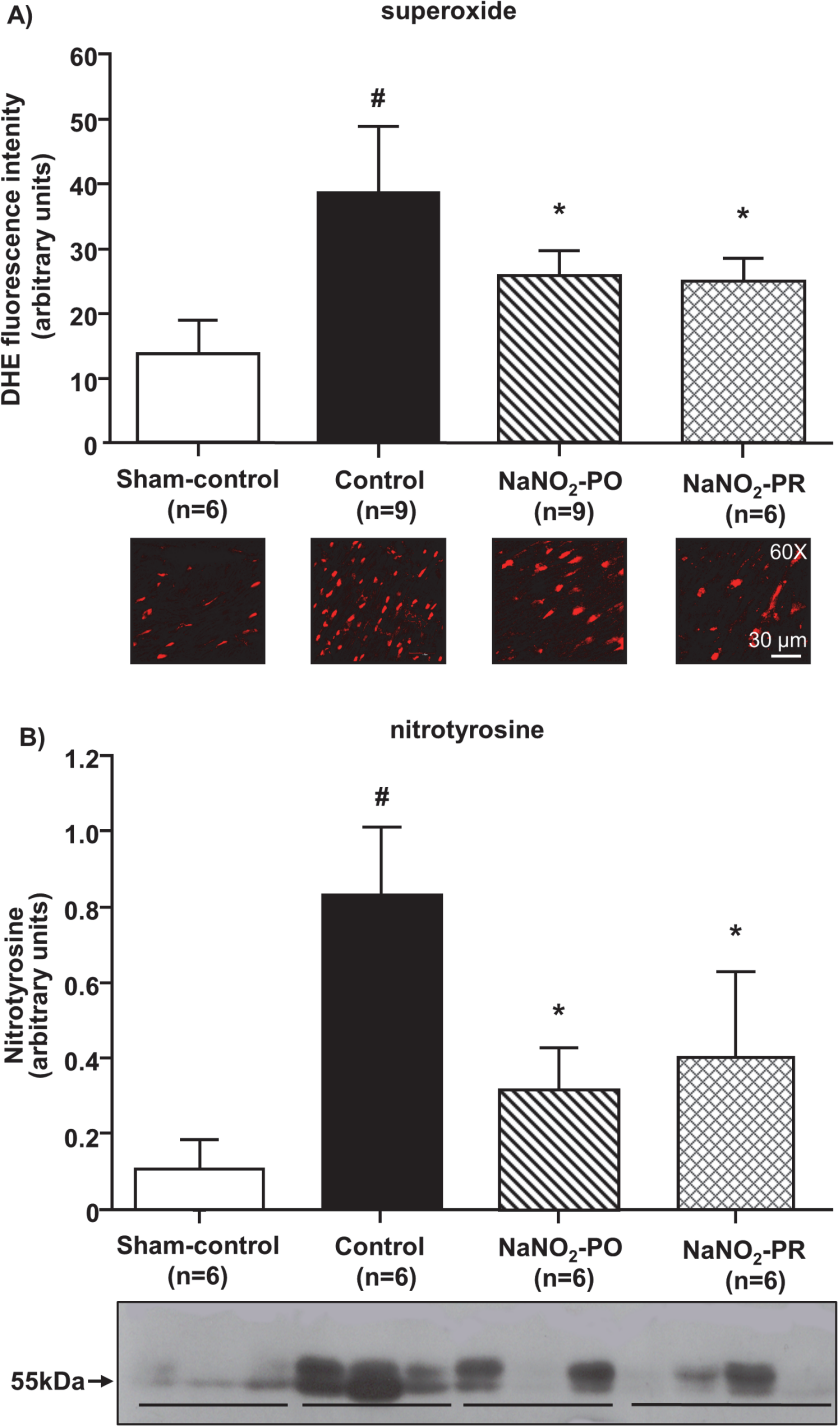
Tissue superoxide (A) and nitrotyrosine (B) production following a 25 min occlusion and reperfusion of the LAD. Compared with the sham-control dogs (instrumented but not subjected to ischaemia and reperfusion; n = 6), there were significant increases in superoxide and nitrotyrosine (NT) formation in controls that had been subjected to ischaemia and reperfusion. Compared with controls, sodium nitrite significantly reduced superoxide and NT production, no matter when the infusion was initiated. Values represent mean ± SEM. ^#^P < 0.05 vs. sham-control dogs, *P < 0.*05* vs. ischaemic controls.

These were determined in all dogs during reperfusion, no matter whether dogs fibrillated or survived (Fig 6). Compared with sham controls (infused only with saline without subjection to ischaemia and reperfusion) 25 min of ischaemia and reperfusion resulted in marked increases in both superoxide (Fig 6A) and nitrotyrosine (Fig 6B) production. These were significantly less marked in those dogs that had been infused with sodium nitrite.

### Effects of sodium nitrite on protein S-nitrosylation and glutathionylation

**Fig 7:**
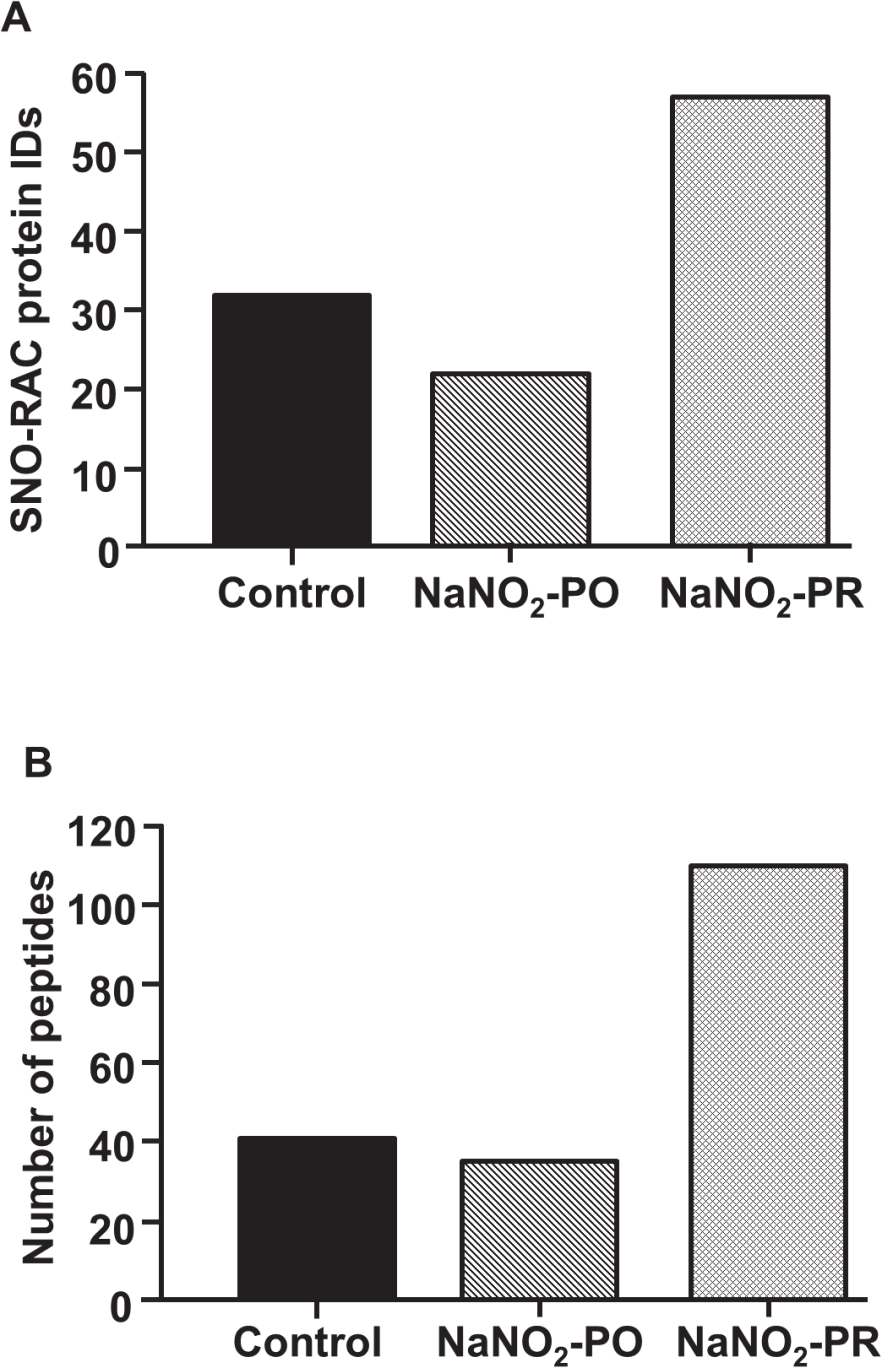
SNO-RAC protein identifications following 25 min ischaemia and reperfusion in control dogs and in dogs infused with sodium nitrite. Compared with controls there was an increase in the number of SNO proteins (A) and peptides (B) in dogs that had been infused with sodium nitrite prior to reperfusion (NaNO_2_-PR group), as determined via liquid chromatography-tandem mass spectrometry for each treatment protocol at a false discovery rate of 5% (n = 4–5 hearts/group).

**Fig 8:**
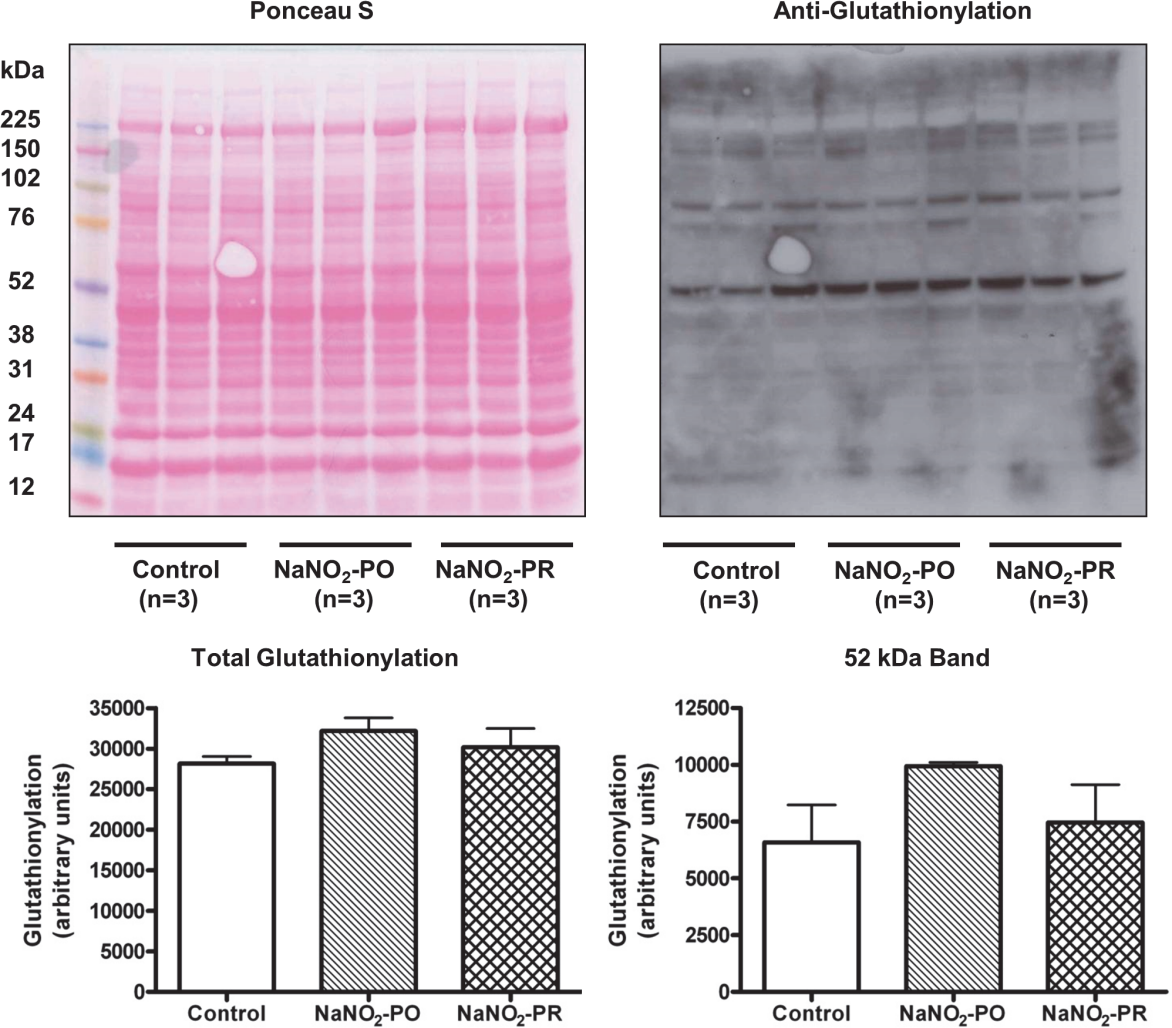
Protein S-glutathionylation levels in control dogs and in dogs infused with sodium nitrite. Compared with controls, there was an increase in protein S-glutathionylation in dogs that had been infused with sodium nitrite prior to occlusion (NaNO_2_-PO group), as determined via Western immunoblot with an anti-glutathionylation antibody. Values represent mean ± SEM. n = 3 hearts/group.

These were determined in tissue samples collected from control and sodium nitrite-treated dogs after 25 min of ischaemia and 2 min reperfusion. The results are summarized in Table 2 and Figs 7 and 8. Compared with controls, the number of proteins and peptides showing cysteine SNO was substantially increased only in dogs that had been infused with sodium nitrite 10 min prior to reperfusion (NaNO_2_-PR group; Fig 7; Table 2). The lack of elevated protein SNO in the NaNO_2_-PO group in comparison to the control and to the NaNO_2_-PR groups led to the examination of protein S-glutathionylation in the same samples. Fig 8 shows that S-glutathionylation is increased in dogs infused with sodium nitrite over a longer period (i.e. 10 min prior to and throughout the 25 min occlusion) especially at the band noted at 52 kDa. Although these changes did not quite meet statistical significance (p = 0.11), the results are consistent with an increase in protein S-glutathionylation in the NaNO_2_-PO group compared with the other two groups.

**Table 2:** Peptides showing cysteine S-Nitrosylation as identified by the SNO-RAC method.

				Control		NaNO_2_-PO		NaNO_2_-PR	
Function	Protein Name	Protein ID	Peptide Sequence	Ratio	Ion Score	Ratio	Ion Score	Ratio	Ion Score
glycolysis	triosephosphate isomerase	P00939	IAVAAQNCYK	1	45	0.15	38	7.19	50
			IIYGGSVTGATCK	1	89	0.94	72	20.74	90
			VPADTEVVCAPPTAYIDFAR	1	63	-	-	2.69	48
	glyceraldehyde-3-phosphate dehydrogenase	Q28554	VPTPNVSVVDLTCR	1	38	0.11	47	1.08	44
			IVSNASCTTNCLAPLAK	1	96	0.71	93	12.49	99
	fructose-bisphosphate aldolase A	P04075	ALANSLACQGK	1	75	0.16†	60	4.12	71
	6-phosphofructokinase	Q0IIG5	LPLMECVQVTK	1*	73	0.21	55	3.49 ‡	6 1
	beta-enolase	Q3ZC09	VNQIGSVTESIQACK	1*	67	-	-	11.40	86
	L-lactate dehydrogenase b chain	P13490	VIGSGCNLDSAR	1	74	0.76	75	41.63‡	80
tricarboxylic acid cycle	malate dehydrogenase, mitochondrial	Q32LG3	GCDVVVIPAGVPR	1	78	1.06	79	10.51‡	64
			EGVVECSFVK	1	45	1.68	45	16.56	49
			TIIPLISQCTPK	1	42	1.47	61	5.08	51
			GYLGPEQLPDCLK	1	72	1.02	69	9.06 ‡	68
	malate dehydrogenase, cytoplasmic	Q3T145	VIVVGNPANTNCLTASK	1	92	0.79	88	21.20‡	60
	pyruvate kinase isozyme M1	P11979	NTGIICTIGPASR	1	68	0.77	49	-	-
	isocitrate dehydrogenase	P54071	VCVQTVESGAMTK	1*	87	0.59	61	-	-
	aconitate hydratase, mitochondrial	P20004	VGLIGSCTNSSYEDMGR	1*	90	-	-	9.03	84
mitochondrial carrier proteins	ADP/ATP translocase 1	P02722	GADIMYTGTVDCWR	1*	67	0.85*	46	12.21‡	52
mitochondrial respiratory chain	ATP synthase subunit epsilon-like protein	Q5VTU8	YSQICAK	1*	45	0.09*	33	-	-
	cytochrome b-c1 complex subunit 1	P31800	LCTSATESEVLR	1*	74	-	-	20.18*	85
cytoskeleton	myosin light chain 3	P08590	ITYGQCGDVLR	1	67	-	-	21.25	75
			LMAGQEDSNGCINYEAFVK	1	63	-	-	0.80	61
	actin, gamma-enteric smooth muscle	Q5E9B5	LCYVALDFENEMATAASSSSLEK	1*	66	0.72	81	5.18*	72
fatty acid metabolism	fatty acid-binding protein, heart	O02772	LILTLTHGSAVCTR	1*	56	-	-	34.13	79
amino acid metabolism	aspartate aminotransferase, mitochondrial	P12344	VGAFTVVCKTCGFDFTGAIEDISK	11	5676	1.570.26	6456	9.633.77	5047
ATP metabolism	creatine kinase M-type	P05123	GYTLPPHCSR	1	41	1.00	34	-	-
	creatine kinase U-type	Q9TTK8	GLSLPPACTR	1*	35	1.91	41	-	-
	60 kDa heat shock protein	P86206	AAVEEGIVLGGGCALLR	1*	69	-	-	0.54*	57
Ca^2+^ storage	sarcoplasmic/endoplasmic reticulum calcium ATPase 2	O46674	VGEATETALTCLVEK	1	103	0.77*	70	3.93	115
others	serum albumin	P49822	YMCENQDSISTK	1	70	0.07†	66	0.77	59
	pancreatic trypsin inhibitor	P00974	AGLCQTFVYGGCR	1*	80	-	-	4.14	67
	hydroxysteroid dehydrogenase-like protein 2	A4FUZ6	LAGCTVFITGASR	1*	86	0.14*	43	5.14*	89

S-nitrosylated proteins and peptides obtained from control and nitrite-treated dogs via SNO-RAC proteomic analysis. For comparison only proteins that were S-nitrosylated in control animals are shown in the table. Peptides were filtered at a false discovery rate of 5%. (C): SNO cysteine residue; (*) peptides were observed in only one SNO-RAC proteomic analysis for a given group. Peptides not detected under the specified condition contain line (-) in the ion score column. Proteome Discoverer v1.3 was used for determining the label free peptide ratio. Values represent mean ± SEM.

^*†*^ *P < 0*.*05* control vs. NaNO_2_-PO,

^*‡*^ *P < 0*.*05* control vs. NaNO_2_-PR group.

### Area at risk

There were no significant differences in the area at risk among the groups. Thus in control dogs (n = 6) the risk area was 41.4 ± 1.0%, in the NaNO_2_-PO group (n = 5) 42.3 ± 1.0% and in the NaNO_2_-PR (n = 5) group 41.8 ± 1.1%.

## Discussion and Conclusions

There is good evidence that nitrite, an oxidative metabolite of NO, represents a natural storage form of NO which under certain conditions, such as low pH and oxygen tension, can readily reduce back to NO [40–42]. Although a protective role for nitrite has been shown under conditions of ischaemia and reperfusion [39, 43, 44], the situation regarding arrhythmias is less clear and has not yet been examined in large animal models. We show here that intravenous administration of sodium nitrite in anaesthetized dogs markedly reduces the severity of arrhythmias that results from a 25 min period of coronary artery occlusion and reperfusion Thus the number of VPBs and VT episodes, the incidence of VT and VF during occlusion were significantly decreased, and survival from the combined ischaemia and reperfusion insult was substantially increased, nonetheless there were great individual variability among dogs in their arrhythmia response to coronary artery occlusion. Interestingly, survival, assessed during the early period of reperfusion (within 2 min), was significantly higher in dogs that were infused with sodium nitrite 10 min prior to reperfusion (92%) than in dogs where nitrite was administered over a longer period of time, i.e. 10 min before and during the entire 25 min of LAD occlusion (50%). Moreover, sodium nitrite also significantly attenuated the ischaemic changes, such as the epicardial ST-segment and TAT. This effect in the NaNO_2_-PR group became apparent shortly after the commencement of the nitrite infusion, when these indices of ischaemia severity started to markedly decline, whereas the phase Ib arrhythmias virtually disappeared. We may draw two conclusions from this finding. First, the effect of nitrite infusion is almost immediate, indicating a rapid conversion of nitrite to NO under ischaemic conditions [43, 44]. Indeed, around 15 min of the occlusion, the ischaemic changes are sufficiently advanced to provide a milieu for the reduction of nitrite to NO. This assumption is also supported by the measurements of NO metabolites (Fig 5). These show that in the NaNO_2_-PR group the plasma nitrite, but not nitrate levels, were elevated over the 10 min period of infusion. It may well be that under these conditions nitrite almost fully reduces to NO, which by replacing the insufficient NO bioavailability due to coronary artery occlusion [22], would lessen the ischaemic changes and the arrhythmias. Second, the high incidence of survivors in the NaNO_2_-PR group suggests that a short period of infusion of nitrite prior to reperfusion is sufficient to increase NO bioavailability and reduce the occurrence of the reperfusion-induced ventricular fibrillation which is common in this canine model when the coronary artery is suddenly reopened [12]. This finding is in accord with the results of Gonzales et al. (2008). They showed in dogs that infusion of nitrite during the last 5 min of ischaemia significantly reduced infarct size and improved recovery of contractile function after reperfusion [39].

There are a number of possible explanations through which NO may influence arrhythmias resulting from ischaemia and reperfusion. Some of these have already been discussed elsewhere [9, 22]. One of the mechanisms to which we paid particular attention in the present study is the regulatory role of NO in the generation of oxidative stress products. Our results showed that sodium nitrite, regardless of the duration of infusion, significantly suppressed the generation of superoxide and peroxynitrite during reperfusion (Fig 6). This is in accord with our [22] and others [45–47] previous findings that an increase in NO bioavailability during ischaemia may suppress the reperfusion-induced generation of ROS. Although there are a number of possible mechanisms through which NO may regulate superoxide production such as by directly acting on or indirectly influencing the components of the electron transport chain (ETC; reviewed e.g. by [46, 48, 49]), the most likely mechanism for the NO-mediated suppressed superoxide generation under conditions of ischaemia/reperfusion is thought to be the posttranslational SNO of mitochondrial complexI [50–53]. Another explanation which would support the marked antiarrhythmic effect of sodium nitrite is the modulatory effect of NO on calcium homeostasis. This also may involve both cGMP-dependent and cGMP-independent mechanisms [29]. The present study has now focused on the cGMP-independent NO-mediated mechanisms, such as the protein S-nitrosylation (SNO), using SNO-RAC proteomic analysis. We found that in dogs given sodium nitrite 10 min prior to reperfusion (NaNO_2_-PR group), the number of SNO proteins and peptides was markedly increased compared with controls subjected to ischaemia and reperfusion without nitrite administration (Fig 7) or with dogs in which nitrite was administerd prior to and during ischaemia (NaNO_2_-PO group). As shown in Table 2 twenty-five proteins exhibited an increase in SNO in the NaNO_2_-PR group. Many of these proteins have been previously shown to undergo increase SNO with preconditioning [26, 30]. Of particular interest, there was an increase in SNO of SERCA2a. We have previously found that SNO of SERCA2a results in an increase in its activity [30], which might lead to better ion homeostasis and contribute to the reduction of arrhythmias. Although we did not observe SNO of other calcium handling proteins, there is evidence that SNO of the L-type Ca^2+^ channels reduces calcium entry [30], or the release of calcium from the sarcuplasmic reticulum is influenced by SNO of the ryanodine receptor [54–56]. These mechanisms may counteract the ischaemia and reperfusion-induced calcium overload and contribute to cardioprotection.

In the NaNO_2_-PR group we also found an increase in SNO of many proteins that are involved in cardiac metabolism and energetics. For example, several proteins involved in glycolysis and in the Krebs cycle showed increased SNO (Table 2). These enzymes are rich in both thiols and metal centers, making them easily react with NO. Although the exact role of SNO in many of these metabolism-related proteins has not yet been well characterized, the results give a support for the regulatory function of this mechanism in glycolysis and energy metabolism by reversible shielding critical cysteins of these enzymes from irreversible oxidation, thereby allowing a more rapid metabolic recovery from an ischaemia/reperfusion insult [23]. It is also hypothesized that the reversible inhibition of the Krebs cycle enzymes would result in slower electron transfer into the ETC with a subsequent reduction in the superoxide production during reperfusion [46].

However, surprisingly, we did not find increased SNO in dogs where nitrite was administered prior to and during occlusion (NaNO_2_-PO group), although the protection against the arrhythmias and ischaemic changes was present, and the generation of oxidative stress products was also significantly suppressed. This conflict requires further considerations. The lack of SNO in the NaNO_2_-PO group might suggest that nitrite is oxidized to nitrate rather than reduced to NO in these hearts. This assumption seems to be supported by the fact that there was a continuous increase in plasma nitrate levels in this group (Fig 5). However, considering that the samples were taken from the coronary sinus which collects blood from both the normal and ischaemic myocardium, it is unlikely that nitrate measured in the plasma is equivalent with nitrate concentrations in the ischaemic tissue and accurately reflects the oxidation/reduction of the infused nitrite. Another explanation for the reduced superoxide and nitrotyrosine production as well as for the obvious antiarrhythmic effect in these dogs might be that when nitrite is infused for a longer period of time the protective effects of NO are predominantly mediated through other mechanisms than SNO. Nitric oxide has many actions, which include both cGMP-dependent and cGMP-independent mechanisms. However, the reactions of NO are influenced by many different factors, such as the local concentration of NO and other molecules that react with NO (e.g. superoxide, heme proteins) or the surrounding milieu in which NO is produced etc. It might well be that the reduction in superoxide production in the NaNO_2_-PO group results primary from other actions of NO. These may include, among the others, the direct interaction of NO with heme proteins, such as soluble guanylyl cyclase, the terminal complex IV cytochrome oxidase [51], the NO mediated inhibition of neutropil superoxide production [57], as well as the NO-dependent activation of antioxidant enzymes [58] and signalling pathways, such as the cGMP/PKG pathway [59].

We also considered another possibility which might explain the lack of SNO in the NaNO_2_-PO group. We supposed that when nitrite is administered over a longer period of time (i.e. prior to and during the entire occlusion interval), NO is generated and primarily promotes protein S- glutathionylation via GSNO. There is evidence that under conditions of continuous NO exposure resulting either from exogenous (administration of NO donors) or endogenous (activation of eNOS/iNOS) sources, NO induces protein S-glutathionylation [60]. This is thought to regulate protein function and particularly under oxidative stress, protects protein thiols from irreversible protein oxidation by providing a “glutathione cap” [60]. If this concept is true, then the protection against arrhythmias in the NaNO_2_-PO group can be explained by increased protein S-glutathionylation rather than protein SNO. To examine this possibility we measured, in the same tissue samples, protein S-glutathionylation. The results show that glutathionylation was somewhat more marked in the NaNO_2_-PO group than in the control and the NaNO_2_-PR groups (Fig 8). Although this change was not statistically significant (p = 0.11), the results indicate an increased number of glutathionylated proteins in this group and support the hypothesis that protein S-glutathionylation might also lead to protection [61, 62].

In summary, our results provide the first evidence that inorganic nitrite, administered in intravenous infusion in a concentration which results in a slight (approximate 7 mmHg) reduction in mean arterial blood pressure without substantially affecting coronary blood flow, markedly reduces the severity of ventricular arrhythmias that result from a 25 min occlusion and reperfusion of the LAD. This antiarrhythmic effect is particularly pronounced when the infusion of nitrite was started 10 min prior to reperfusion; in this case, survival from the combined ischaemia and reperfusion insult was 92%, compared with 0% in the controls and 50% in dogs in which nitrite was infused prior to and during the occlusion. Interestingly, the protective effect of nitrite was associated with increased protein SNO only when nitrite had been administered 10 min prior to reperfusion; the infusion of nitrite over a longer period seemed to prefer protein S-glutathionylation with subsequent protection against the serious consequences of the ischaemia and reperfusion. We propose that the marked antiarrhythmic effects of nitrite administration is associated with increased NO bioavailability during occlusion and the subsequent reduction in the effect of oxidative stress on reperfusion via those NO mediated mechanisms which reversibly protect proteins from oxidation during the early ‘critical’ phase of reperfusion.

## Supporting Information

S1 Dataset All the data presented in this paper are provided as an online supplement (S1_Dataset.xls). (XLS)
